# Emergence of a New Lineage of Dengue Virus Type 2 Identified in Travelers Entering Western Australia from Indonesia, 2010-2012

**DOI:** 10.1371/journal.pntd.0003442

**Published:** 2015-01-30

**Authors:** Timo Ernst, Suzi McCarthy, Glenys Chidlow, Dagwin Luang-Suarkia, Edward C. Holmes, David W. Smith, Allison Imrie

**Affiliations:** 1 School of Pathology and Laboratory Medicine, University of Western Australia, Nedlands, Western Australia, Australia; 2 PathWest Laboratory Medicine WA, Nedlands, Western Australia, Australia; 3 Papua New Guinea Institute of Medical Research, Goroka, Papua New Guinea; 4 Marie Bashir Institute for Emerging Diseases & Biosecurity, Charles Perkins Centre, School of Biological Sciences and Medical School, The University of Sydney, Sydney, New South Wales, Australia; Baylor College of Medicine, UNITED STATES

## Abstract

Dengue virus (DENV) transmission is ubiquitous throughout the tropics. More than 70% of the current global dengue disease burden is borne by people who live in the Asia-Pacific region. We sequenced the E gene of DENV isolated from travellers entering Western Australia between 2010–2012, most of whom visited Indonesia, and identified a diverse array of DENV1-4, including multiple co-circulating viral lineages. Most viruses were closely related to lineages known to have circulated in Indonesia for some time, indicating that this geographic region serves as a major hub for dengue genetic diversity. Most notably, we identified a new lineage of DENV-2 (Cosmopolitan genotype) that emerged in Bali in 2011–2012. The spread of this lineage should clearly be monitored. Surveillance of symptomatic returned travellers provides important and timely information on circulating DENV serotypes and genotypes, and can reveal the herald wave of dengue and other emerging infectious diseases.

## Author Summary

Dengue is currently the most rapidly spreading mosquito-borne viral disease of humans, and is endemic in most tropical and sub-tropical countries. An estimated 390 million infections occur annually, and over 70% of the current global dengue disease burden is borne by people who live in Southeast Asia and the Western Pacific region. DENV serotype and genotype data is lacking in many parts of this region, limiting our attempts to understand the observed patterns of hyperendemicity and disease severity. Many countries in the Southeast Asia and Western Pacific region are popular tourist destinations, and dengue has been identified as a cause of travel-related illness in people returning from endemic countries. We sequenced the E gene of DENV isolated from travellers returning to Western Australia from 7 countries throughout Asia between 2010 and 2012. The majority of DENV originated in Indonesia, predominantly Bali, a popular travel destination for Australians. We identified hyperendemic transmission of all four DENV serotypes in Bali in 2010; circulating DENV included dominant local strains which had circulated for several years in Indonesia and Singapore, as well as strains more recently introduced into Bali from other countries in the region. Finally, we show the emergence of a new lineage of DENV2 (Cosmopolitan genotype) in 2011–2012, which should be monitored. Travellers may act as sentinels and provide important information on DENV genotypes and linages circulating in countries where locally generated detailed genetic data may not be available.

## Introduction

Dengue is the most rapidly spreading mosquito-borne viral disease of humans. An estimated 390 million dengue virus infections occur annually, of which approximately one quarter result in clinical disease [1]. Dengue transmission is predicted to be ubiquitous throughout the tropics, with the highest incidence rates in Asia and the Americas. More than 70% of the current global dengue disease burden is borne by people who live in Southeast Asia and the Western Pacific region [2], where dengue incidence has increased consistently since 2000.

Dengue is caused by infection with any one of four antigenically distinct dengue viruses (DENV), single-stranded positive-sense RNA viruses that form their own antigenic complex within the family *Flaviviridae*. There are four DENV serotypes (DENV1–4), and each DENV serotype can further be subdivided into distinct genotypes, and lineages within the genotypes, largely based on phylogenetic analysis of envelope (E) gene sequences [3–6]. Accordingly, analyses of E gene sequences have revealed that DENV-1 and DENV-2 can be divided into five and six genotypes, respectively, and DENV-3 and DENV-4 into four genotypes, including the sylvatic lineages found in non-human primates.

Previous studies of DENV molecular epidemiology have revealed relatively stable genotypic compositions within individual localities, punctuated by occasional lineage turnover [7]. Although the causes of these lineage replacement events are often opaque, in some cases they have been shown to reflect underlying fitness differences. For example, studies in Vietnam showed that between 1995 and 2009 the dominant Asian /American lineage of DENV-2 was replaced by the Asian I lineage [8]. This displacement was thought to be driven by higher viremia levels in patients infected with the Asian I lineage, potentially leading to increased human-mosquito transmission. In addition, the introduction of new lineages may be associated with higher rates of severe disease, as was shown in the Caribbean in 1981 when the first appearance of the Asian/American [9] lineage of DENV-2 coincided with the first recorded DHF-associated epidemics in the region. As a consequence, it is critical to monitor the appearance and spread of novel lineages, particularly those that are often associated with severe dengue disease.

Dengue transmission in the Asia-Pacific region, including Australia, has been documented since the 1940s, when the four prototype DENV were first identified in Hawaii (DENV-1_Hawaii_, 1944), New Guinea (DENV-2_NGC_, 1944), and the Philippines (DENV-3_H-87_ and DENV-4_H-421_, 1956) [10–12]. Marked increases in DENV-1–4 transmission have been documented throughout the region over the past three decades and increasing travel within the region has supported introduction of DENV from epidemic areas to countries many thousands of kilometres away [13–16]. Despite the burden of dengue in the Asia-Pacific region, data on which serotypes and genotypes are circulating in many parts of the region are lacking [17], limiting our attempts to understand the mechanisms governing the observed patterns of disease severity and hyperendemicity.

To identify and characterize prevailing serotypes and genotypes in the Asian region we undertook an analysis of DENV in symptomatic infected travellers entering Western Australia, and in doing so identified the emergence of a new lineage of DENV-2 in Bali, Indonesia.

## Materials and Methods

### Viruses

**Figure 1:**
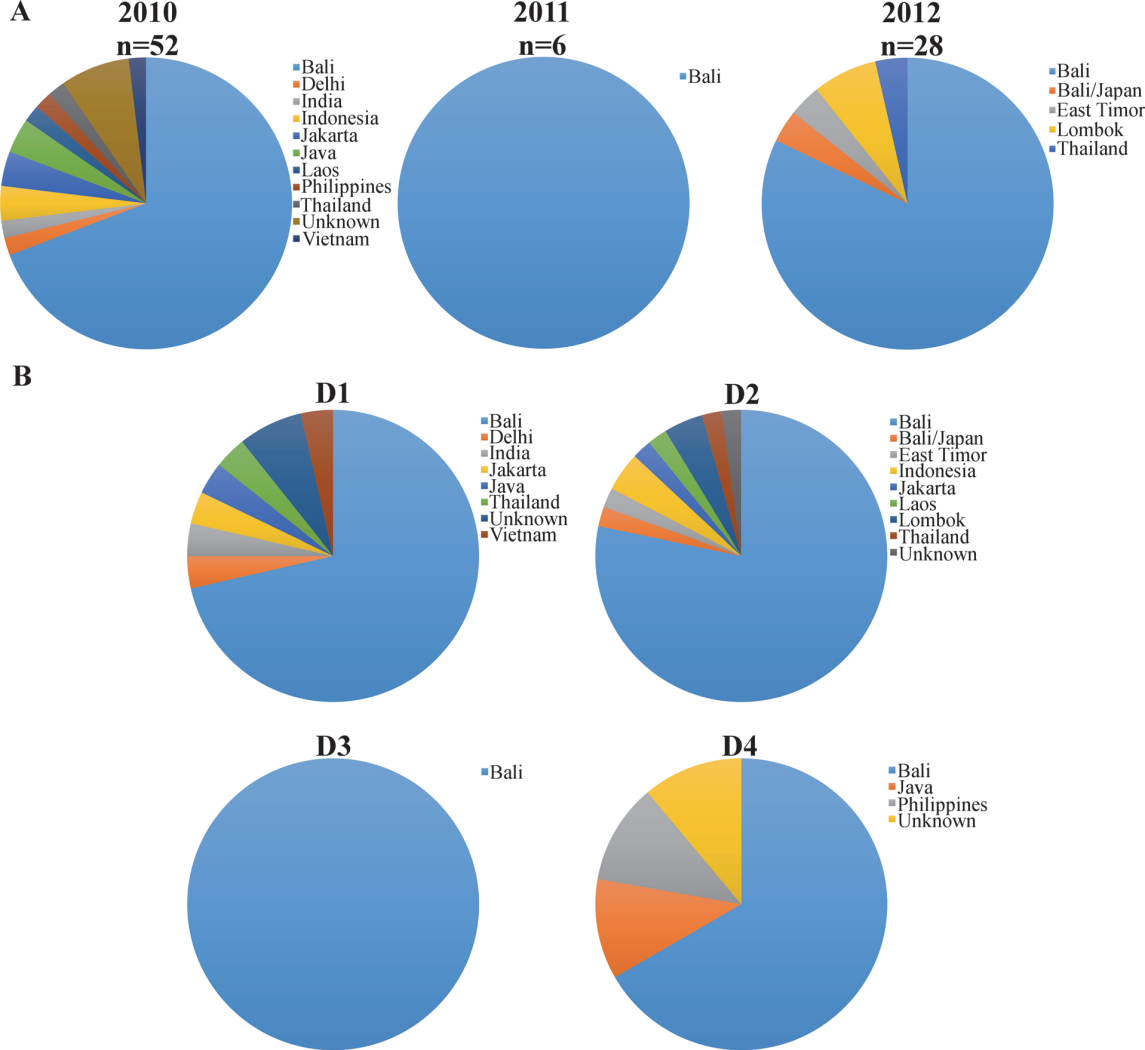
Origin and diversity of dengue viruses isolated from travelers entering Western Australia during 2010–2012. (A) Geographic origins of all imported DENV; (B) Geographic origin of individual DENV serotypes.

DENV was isolated from de-identified acute phase serum samples submitted to Pathwest Laboratory Medicine WA for diagnostic testing. These serum samples were stored at -20°C until used. Virus isolation was attempted on 313 serum samples. Briefly, 100μl of NS1 antigen-positive serum (Platelia Dengue NS1 Antigen ELISA kit; Bio-Rad, Australia) was inoculated onto Vero cells in 5.5ml cell culture tubes and cell culture supernatants were harvested after 2 or 3 blind passages. Briefly, 100μl of serum was inoculated onto a Vero cell monolayer in minimal media and incubated overnight. On the following day the inoculum was removed and 3ml of DMEM with 2% FBS was added to the cells, and the culture was maintained for 7 days at 37°C with 5% CO_2_. Successful virus isolation was identified by NS1 antigen ELISA. A total of 86 viruses (27.5%) were successfully isolated, from seven different countries: 74 isolates from Indonesia, of which 66 originated in Bali; two from Thailand; two from India, and one each from Laos, Philippines, Timor-Leste, and Vietnam (an additional four viruses were from unspecified countries). One person reported travel to Bali and Japan. Fifty-one viruses were isolated from samples submitted in 2010, six from 2011, and 29 from 2012 (Table 1; Fig. 1). Sample data were analyzed anonymously. This project was approved by the University of Western Australia Human Research Ethics Committee.

**Table 1:** Origin and phylogenetic placement of DENV sequences from travelers entering Western Australia.

Sequence ID	Entering WA From	Date of Collection	Genotype, Lineage
DENV-1			
D1/IDN/Bali_004/2010	Bali	10/28/2010	Genotype II, Lineage 2
D1/IDN/Bali_005/2010	Bali	11/18/2010	Genotype II, Lineage 2
D1/IDN/Bali_009/2010	Bali	4/27/2010	Genotype I, Lineage 14
D1/IND/Delhi_011/2010	Delhi	10/21/2010	Genotype III, Lineage 1
D1/IDN/Bali_012/2010	Bali	9/19/2010	Genotype II, Lineage 6
D1/IDN/Bali_013/2010	Bali	11/2/2010	Genotype II, Lineage 2
D1/IDN/Bali_014/2010	Bali	5/5/2010	Genotype I, Lineage 13
D1/IDN/Bali_015/2010	Bali	8/6/2010	Genotype II, Lineage 2
D1/IND/022/2010	India	9/24/2010	Genotype III, Lineage 1
D1/IDN/Java_023/2010	Java	11/16/2010	Genotype II, Lineage 2
D1/IDN/Bali_024/2010	Bali	8/12/2010	Genotype I, Lineage 14
D1/IDN/Bali_030/2010	Bali	12/22/2010	Genotype II, Lineage 2
D1/031/2010	N/A	9/6/2010	Genotype I, Lineage 10
D1/IDN/Bali_032/2010	Bali	12/21/2010	Genotype II, Lineage 2
D1/IDN/Bali_033/2010	Bali	5/27/2010	Genotype I, Lineage 14
D1/IDN/Bali_034/2010	Bali	4/28/2010	Genotype II, Lineage 4
D1/IDN/Bali_037/2010	Bali	8/19/2010	Genotype I, Lineage 12
D1/IDN/Bali_038/2010	Bali	10/18/2010	Genotype I, Lineage 13
D1/IDN/Bali_039/2010	Bali	12/29/2010	Genotype II, Lineage 3
D1/040/2010	N/A	6/9/2010	Genotype I, Lineage 8
D1/THA/41/2010	Thailand	12/16/2010	Genotype I, Lineage 9
D1/VNM/042/2010	Vietnam	5/8/2010	Genotype II, Lineage 6
D1/IDN/Jakarta_048/2010	Jakarta	8/25/2010	Genotype II, Lineage 5
D1/IDN/Bali_055/2010	Bali	4/22/2010	Genotype II, Lineage 4
D1/IDN/Bali_068/2011	Bali	8/15/2011	Genotype 1, Lineage 14
D1/IDN/Bali_071/2011	Bali	3/1/2011	Genotype I, Lineage 13
D1/IDN/Bali_072/2011	Bali	3/19/2011	Genotype I, Lineage 11
D1/IDN/Bali_073/2011	Bali	4/8/2011	Genotype I, Lineage 11
DENV-2			
D2/IDN/Bali_010/2010	Bali	8/5/2010	Cosmopolitan, Lineage 1
D2/IDN/Bali_020/2010	Bali	10/16/2010	Cosmopolitan, Lineage 5
D2/IDN/Bali_021/2010	Bali	8/6/2010	Cosmopolitan, Lineage 1
D2/IDN/Bali_025/2010	Bali	10/16/2010	Cosmopolitan, Lineage 5
D2/IDN/Bali_035/2010	Bali	12/26/2010	Cosmopolitan, Lineage 8
D2/LAO/043/2010	Laos	10/8/2010	Asian I, Lineage 9
D2/IDN/044/2010	Indonesia	6/10/2010	Cosmopolitan, Lineage 1
D2/IDN/045/2010	Indonesia	6/6/2010	Cosmopolitan, Lineage 3
D2/046/2010	N/A	5/29/2010	Cosmopolitan, Lineage 7
D2/IDN/Bali_047/2010	Bali	5/7/2010	Cosmopolitan, Lineage 3
D2/IDN/Bali_049/2010	Bali	9/3/2010	Cosmopolitan, Lineage 5
D2/IDN/Bali_050/2010	Bali	6/10/2010	Cosmopolitan, Lineage 5
D2/IDN/Bali_051/2010	Bali	4/22/2010	Cosmopolitan, Lineage 1
D2/IDN/Bali_052/2010	Bali	8/6/2010	Cosmopolitan, Lineage 6
D2/IDN/Bali_057/2010	Bali	4/19/2010	Cosmopolitan, Lineage 2
D2/IDN/Bali_059/2010	Bali	11/2/2010	Cosmopolitan, Lineage 7
D2/IDN/Jakarta_060/2010	Jakarta	10/18/2010	Cosmopolitan, Lineage 5
D2/IDN/Bali_075/2011	Bali	12/14/2011	Cosmopolitan, Lineage 4
D2/IDN/Bali_076/2012	Bali	1/16/2012	Cosmopolitan, Lineage 4
D2/IDN/Bali_077/2012	Bali	1/17/2012	Cosmopolitan, Lineage 4
D2/TLS/Timor_078/2012	Timor Leste	1/21/2012	Cosmopolitan, Lineage 4
D2/IDN/Bali_079/2012	Bali	1/25/2012	Cosmopolitan, Lineage 4
D2/IDN/Bali_080/2012	Bali	1/25/2012	Cosmopolitan, Lineage 4
D2/IDN/Bali_081/2012	Bali	1/27/2012	Cosmopolitan, Lineage 4
D2/IDN/Bali_082/2012	Bali	1/29/2012	Cosmopolitan, Lineage 4
D2/IDN/Bali_084/2012	Bali	1/30/2012	Cosmopolitan, Lineage 4
D2/THA/086/2012	Thailand	1/31/2012	Cosmopolitan, Lineage 4
D2/IDN/Lombok_087/2012	Lombok	2/1/2012	Cosmopolitan, Lineage 4
D2/IDN/Bali_088/2012	Bali	2/2/2012	Cosmopolitan, Lineage 4
D2/IDN/Bali_089/2012	Bali	2/2/2012	Cosmopolitan, Lineage 4
D2/IDN/Bali_090/2012	Bali	2/3/2012	Cosmopolitan, Lineage 4
D2/IDN/Lombok_091/2012	Lombok	2/3/2012	Cosmopolitan, Lineage 4
D2/IDN/Bali_092/2012	Bali	2/6/2012	Cosmopolitan, Lineage 4
D2/IDN/Bali_094/2012	Bali	2/10/2012	Cosmopolitan, Lineage 4
D2/IDN/Bali_095/2012	Bali	2/13/2012	Cosmopolitan, Lineage 4
D2/IDN/Bali_096/2012	Bali	2/13/2012	Cosmopolitan, Lineage 4
D2/IDN/Bali_097/2012	Bali	2/13/2012	Cosmopolitan, Lineage 4
D2/IDN/Bali_098/2012	Bali	2/14/2012	Cosmopolitan, Lineage 4
D2/IDN/Bali_100/2012	Bali	2/15/2012	Cosmopolitan, Lineage 4
D2/IDN/Bali_102/2012	Bali	2/24/2012	Cosmopolitan, Lineage 4
D2/IDN/Bali_103/2012	Bali/Japan	2/24/2012	Cosmopolitan, Lineage 4
D2/IDN/Bali_104/2012	Bali	2/27/2012	Cosmopolitan, Lineage 4
D2/IDN/Bali_105/2012	Bali	2/28/2012	Cosmopolitan, Lineage 4
D2/IDN/Bali_106/2012	Bali	3/5/2012	Cosmopolitan, Lineage 4
D2/IDN/Bali_107/2012	Bali	3/6/2012	Cosmopolitan, Lineage 4
D2/IDN/Bali_108/2012	Bali	2/6/2012	Cosmopolitan, Lineage 4
DENV-3			
D3/IDN/Bali_007/2010	Bali	12/30/2010	Genotype I, Lineage 3
D3/IDN/Bali_054/2010	Bali	3/6/2010	Genotype I, Lineage 1
D3/IDN/Bali_056/2010	Bali	9/21/2010	Genotype I, Lineage 2
DENV-4			
D4/IDN/Java_002/2010	Java	10/7/2010	Genotype III, Lineage 4
D4/PHL/006/2010	Philippines	2/8/2010	Genotype III, Lineage 5
D4/IDN/Bali_036/2010	Bali	3/3/2010	Genotype III, Lineage 1
D4/IDN/Bali_062/2010	Bali	11/13/2010	Genotype III, Lineage 2
D4/IDN/Bali_063/2010	Bali	11/11/2010	Genotype III, Lineage 2
D4/IDN/Bali_064/2010	Bali	11/8/2010	Genotype III, Lineage 3
D4/IDN/Bali_065/2010	Bali	10/18/2010	Genotype III, Lineage 3
D4/ 066/2010	N/A	5/4/2010	Genotype III, Lineage 2
D4/IDN/Bali_069/2011	Bali	9/8/2011	Genotype III, Lineage 2

### RNA extraction and sequencing

Viral RNA was extracted from 140 μl of culture supernatant using QIAmp viral RNA Mini kits (Qiagen), according to the manufacturer’s instructions. cDNA was synthesized from extracted RNA using SuperScript III First-Strand Synthesis System for RT-PCR (Invitrogen) as per the manufacturer’s instructions. DENV serotype was confirmed by RT-PCR using serotype-specific primers (Table 2), and the LongRange PCR Kit (Qiagen) (thermocycling conditions are available on request). To remove excess oligonucleotide primers and dNTPs, PCR products were prepared for sequencing using Exo-SAP-IT following the manufacturer’s protocol (USB Corporation, Cleveland USA). The nucleotide sequences were determined with template-specific primers (Table 2) using fluorescence-based cycle sequencing conditions (BigDye Terminator v3.1 Cycle Sequencing kit, AB, Foster City USA). All incubation steps were completed in thermal cyclers (AB2720 thermal cycler, AB, Foster City USA). Unincorporated dye terminators were removed from sequencing reactions using gel-filtration following the manufacturer’s protocol (DyeEx 2.0 Spin Kit, GmbH, Germany). Sequencing was performed on a 16-capillary genetic analyzer (3130xl Genetic Analyzer, AB, Foster City USA). Chromatogram editing (Chromas v2.3, Technelysium Pty Ltd) and sequence alignment (Bioedit, TA Hall software, and the Lasergene software of DNA Star (DNASTAR, Madison USA)) were undertaken by comparison to sequence information in GenBank. All sequences have been deposited in GenBank and assigned accession numbers KM216664-KM216747 and KM222447-KM222448.

**Table 2:** Primers used in PCR amplification and sequencing reactions.

DENV serotype	Primer*	Primer pair	Fragment size (bp)	Primer sequence (5’-3’)
1	D1–760F†	1	840	AAC GTG GAT GTC CTC TGA AGG
	D1–1600R†			CGA GGT CCA AGG CAG TG
	D1–1418F†	2	1182	GCA ACC ATA ACA CCT CAA
	D1–2600R†			TGG CTG ATC GAA TTC CAC AC
	D1–1220F‡			TTT GTG GAC AGA GGC TGG G
	D1–1559F‡			CAC AAA CAA TGG TTT CTA GAC TTA C
	D1–1948F‡			GAC CCA AGA TGA GAA AGG AGT
	D1–1240R‡			TGC CCC AGC CTC TGT CCA C
	D1–1424R‡			GAC GTA GGA GCT TGA GGT GTT AT
	D1–1868R‡			CAT GCT GGG TCT CAG CCA C
2	D2–789F†	1	1131	GAA ACA TGC CCA GAG AAT TGA AAC T
	D2–1920R†			CCC TTC ATA TTG TAC TCT GAT AAC TAT TGT TCC
	D2–1547F†	2	852	AAG CTT GGC TGG TGC ACA GGC AAT GGT T
	D2–2537R†			GGG GAT TCT GGT TGG AAC TTG TAT TGT TCT GTC C
	D2–1136F§			ACA CAA CAA CAG CAT CTC GCT
	D2–1499R§			CTC GGA GAG CAT TCC ATC GT
3	D3–291F†	1	1528	TGG CTA GAT GGG GTA CCT TC
	D3–1819R†			CAT CCC TTT GAG TTT CAA TTT GTC CAT
	D3–1685F†	2	865	CTA GGA TCT CAA GAA GGA GCA ATG CA
	D3–2550R†			ATG GCT GTT GCC ACT CTT TTG GGG GA
	D3–875F§			GGG AAA CGG TTG TGG TTT GT
	D3–1277R§			AAG AAG CTC TTT CTT GTT CCA GGT T
4	D4–742F†	1	1096	TGG GAT TGG AAA CAA GAG CTG AGA CAT GGA TGT C
	D4–1838R†			CGT GTA TGA CAT TCC CTT GAT TCT CAA TTT CTC CA
	D4–1569F†	2	970	CAA TGG TTT TTG GAC CTA CCT CTA CCA TGG
	D4–2539R†			GGG GAC TCT GGT TGA AAT TTG TAC TGT TCT GTC CA

*F indicates forward primer orientation, R indicates reverse primer orientation.

†Primer used for both PCR and sequencing reactions (26)

‡Sequencing primer [27]

§Sequencing primer (designed by Dr. Glenys Chidlow)

### Phylogenetic analysis

To put the evolution of the dengue viruses circulating in travellers returning to Australia into the context of those circulating more widely, and particularly in Asian countries, all E gene sequences of human DENV-1 to DENV-4 were downloaded from GenBank and utilized as a comparison data set. DENV sequences associated with non-human primates (i.e. from the sylvatic cycle), those with excessively long branches such that they are likely to reflect sequencing error, and obvious laboratory contaminants (i.e. where there is little measurable evolution over multiple years, such as multiple copies of the DENV-2 strain New Guinea C) were excluded. Combined with the viruses isolated from travellers to Australia in the present study, this resulted in data sets of the following size that could easily be visually aligned and subjected to phylogenetic analysis: DENV-1 = 2289 taxa, 1485 nt; DENV-2 = 2263 taxa, 1485 nt, DENV-3 = 1256 taxa, 1479 nt, DENV-4 = 670 taxa, 1485 nt.

Phylogenetic trees for each of the four serotypes were estimated using the maximum likelihood (ML) method available in the RAxML program [18] and assuming the GTRGAMMA substitution model (i.e. the general time-reversible nucleotide substitution model), combined with a rapid bootstrap (100 replicates) procedure. The resultant phylogenies were visualized in FigTree (kindly provided by Dr. Andrew Rambaut; http://tree.bio.ed.ac.uk/software/figtree/). The number of phylogenetically distinct lineages of viruses sampled from Australian travellers were identified by a combination of visual inspection (i.e. the detection of phylogenetically distinct groups of sequences associated with travellers) and by the level of bootstrap support, with bootstrap >70% indicative of strong support for groupings of viruses sampled from travellers.

## Results

### Origin of imported DENV

To obtain a snapshot of the diversity of DENV circulating in the South-East Asian region and being imported into Australia we assessed the genetic diversity of DENV isolated from travelers entering Western Australia between March 2010 and March 2012. Accordingly, we noted that the majority of DENV originated in Indonesia, predominantly Bali. This was not unexpected as approximately 80% of dengue cases notified to the Communicable Disease Division of the Western Australian Department of Health in 2010–2012 originated in Bali [19]. Due to the high number of imported cases, Western Australia notifies the most dengue cases among the eight Australian States and Territories despite having only 10% of the Australian population and no endogenous dengue transmission, due to absence of the mosquito vectors.

### DENV diversity

**Figure 2:**
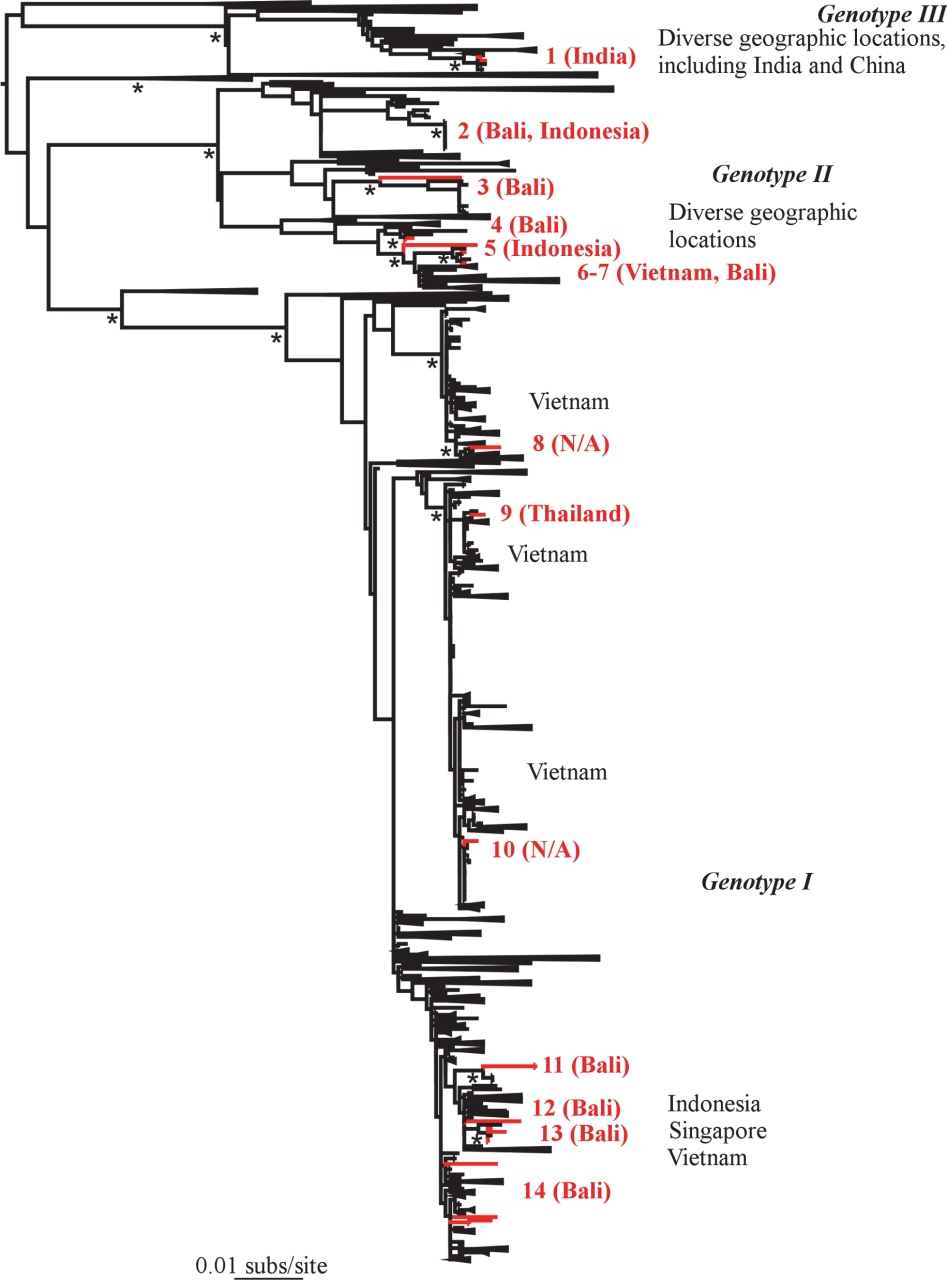
Phylogenetic tree of 2289 DENV-1 E sequences including 28 from travelers to Australia. Because of the very large numbers of sequences involved, where possible phylogenetic clusters have been ‘collapsed’ (such they are they depicted as triangles) to reveal the positions of the Australian viruses in more detail. Major genotypes and geographic locations of sequences are indicated. Those viruses sampled from travellers to Australia are colored red, numbered according to phylogenetic lineage (Table 1), with the likely place of infection noted. Bootstrap support values (>70%) are shown for key nodes and all horizontal branch lengths are drawn to a scale of nucleotide substitutions per site. The tree is mid-point rooted for clarity only.

**Figure 3:**
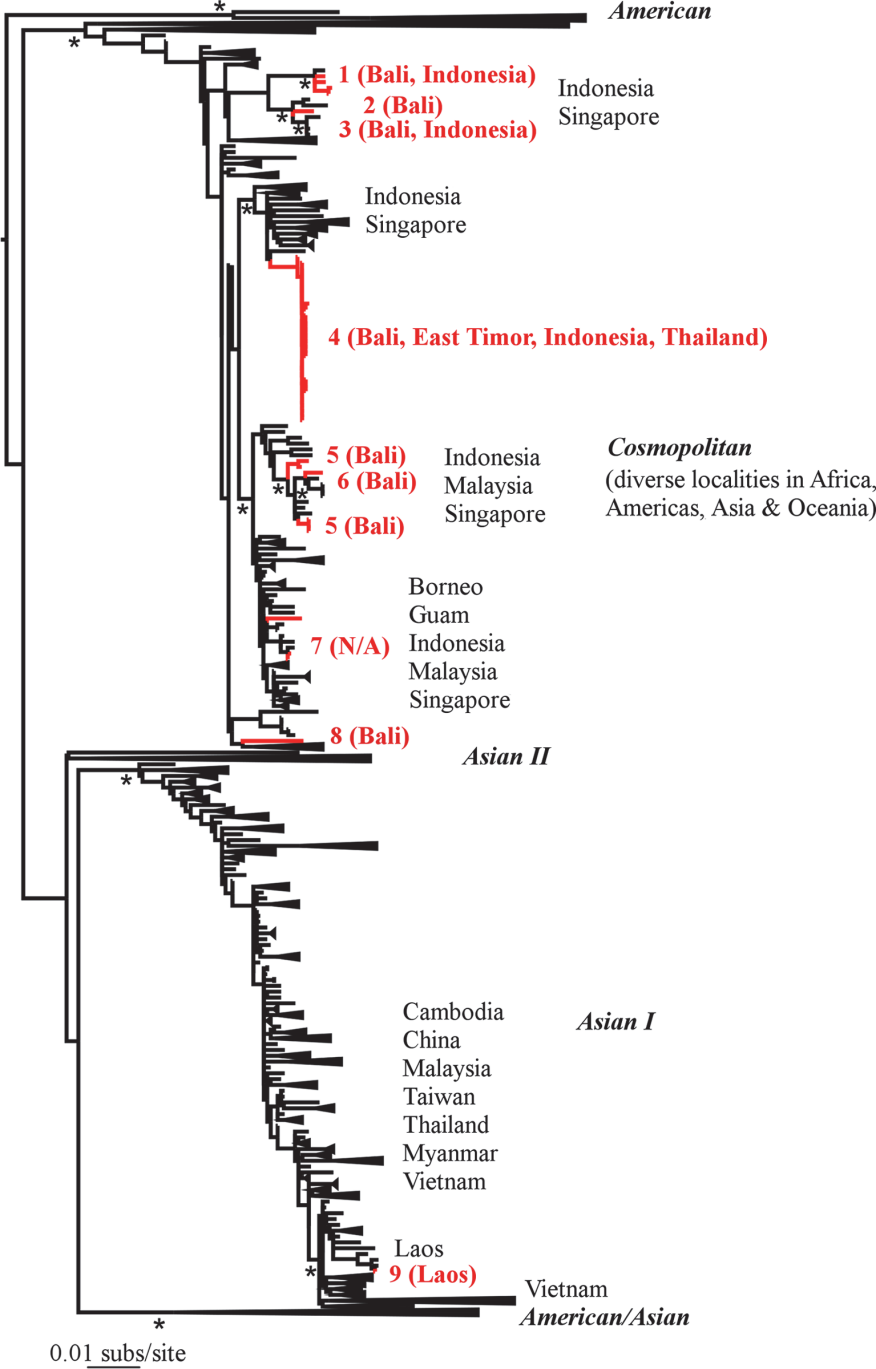
Phylogenetic tree of 2263 DENV-2 E sequences including 46 from travellers to Australia. Other features are as they are described in Fig. 2.

**Figure 4:**
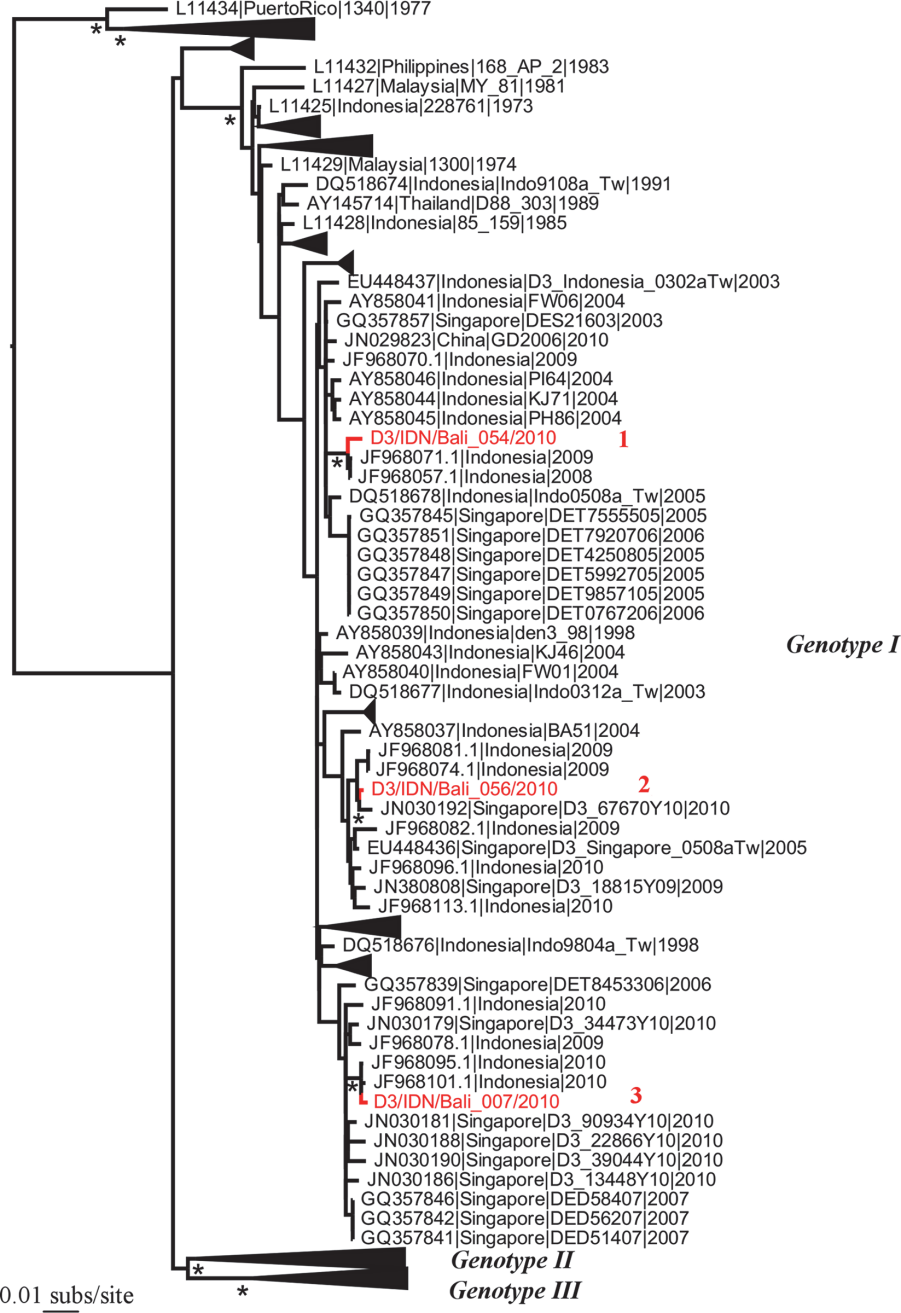
Phylogenetic tree of 1256 DENV-3 E sequences including three from travellers to Australia. Other features are as they are described in Fig. 2.

**Figure 5:**
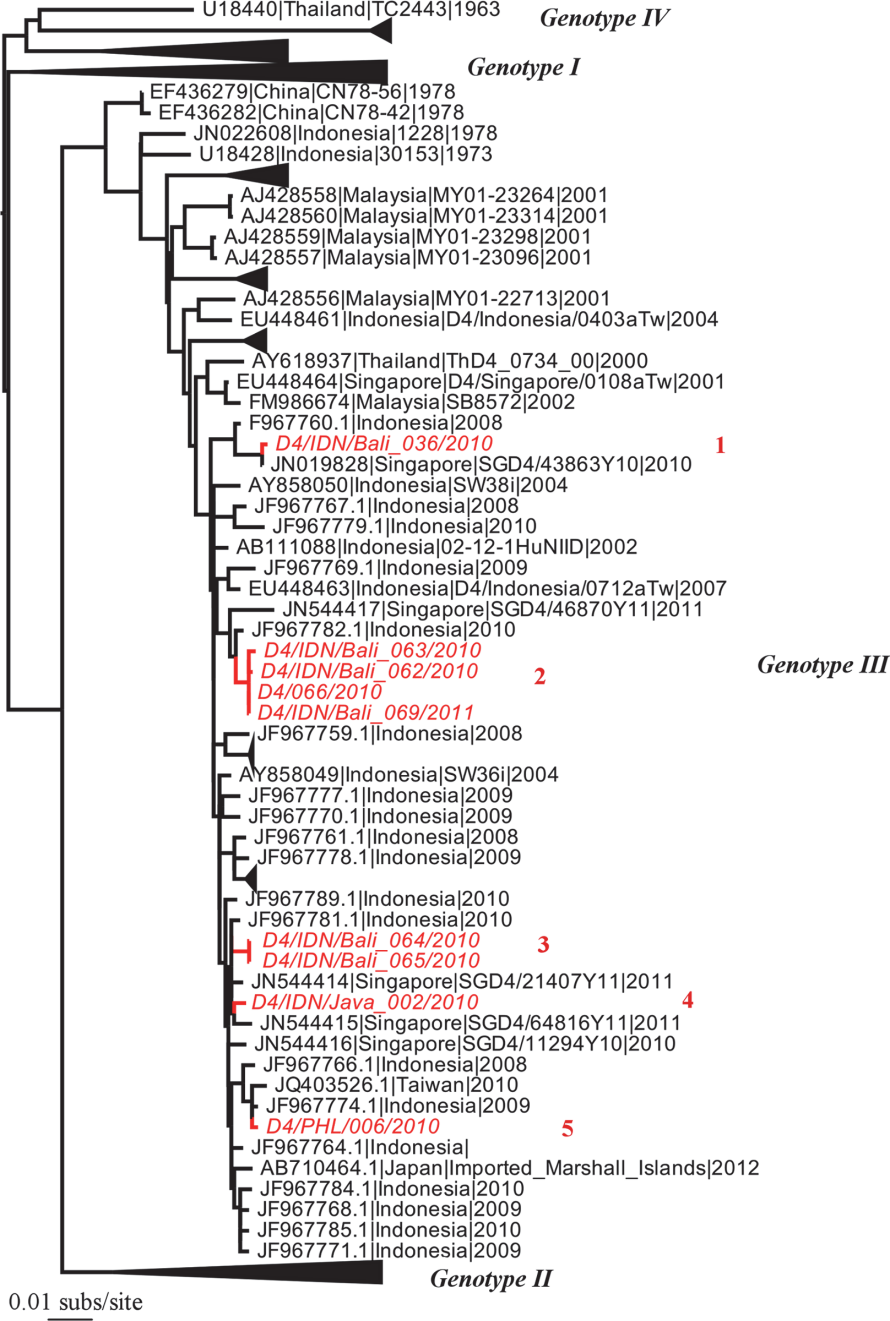
Phylogenetic tree of 670 DENV-4 E sequences including nine from travellers to Australia. Other features are as they are described in Fig. 2.

Our phylogenetic analysis of the four DENV serotypes revealed a remarkable diversity of viruses in Australian travellers, including the co-circulation of multiple genetically diverse viral lineages (Table 1; Figs. 1–5). With a few interesting exceptions, the viruses sampled from travelers to Australia were generally closely related to those previously circulating in regions of Indonesia, particularly Bali. We now describe each serotype in turn.

### DENV-1

Our analysis of DENV-1 comprised a total of 28 sequences from travelers entering Western Australia with clinical dengue who were sampled between April 2010 and August 2011. These viruses fell into 14 phylogenetically distinct lineages in three DENV-1 genotypes (Fig. 2), making this the most genetically diverse of all the four serotypes found in this study. As expected, the majority of these sequences were closely related to those previously documented to be circulating in Bali and Indonesia, falling into genotypes I and II of DENV-1 that are common in South-East Asia, and which suggests that these lineages are the dominant regional variants. The only exceptions were two travellers returning from India who, notably, possessed genotype III strains most closely related to viruses circulating in China (lineage 1). Similarly, the lineage 2 (genotype II) viruses of Balinese origin were most closely related to viruses from China (and which share identical E gene sequences). These phylogenetic patterns are compatible with viral traffic between China, India and Bali that merits further investigation. Finally, it was also notable that a number of lineages co-circulated in the Balinese source population. Specifically, lineages 2, 6, 13 and 14 co-circulated for a period during 2010, while lineages 11, 13 and 14 co-circulated during 2011. Indeed, it is striking that lineage 14 persisted for over a year (April 2010–August 2011).

### DENV-2

The biggest data set (n = 46) of traveler sequences available here were from DENV-2. With the exception of one sequence, all these viruses were from the Cosmopolitan genotype that has been sampled from a wide range of geographic localities including those in Africa, the Americas, Asia (including Indonesia, Malaysia, Myanmar, Thailand, Singapore) and Oceania (Fig. 3). The single exception (lineage 9) was sampled from Laos and fell within the Asian I genotype, and closely related to a number of other viruses from Laos. Hence, these data provide some support for the existence of a major phylogeographic split, with the Asian I genotype largely sampled in the northern part of South-Asia (including Myanmar, Thailand, Vietnam), and the Cosmopolitan genotype being sampled in more southerly regions. Including the single sequence from Laos, our phylogenetic analysis provided evidence for nine separate lineages, which likely represent separate introductions, into Australian travelers. Again, these introductions largely seem to have their origins in Bali, as they were all closely related to viral lineages previously circulating in either Bali or other regions of Indonesia. Perhaps the most notable phylogenetic pattern was the presence of a major cluster of closely related sequences in Bali (lineage 4) found in patients sampled between December 2011 and March 2012, and which was the most recently circulating lineage detected in this study. Such clustering is indicative of a major local outbreak in Bali which has also affected other parts of Indonesia (Lombok), Timor-Leste and Thailand as the cluster contains sequences sampled from travelers entering from these localities. In addition, given the very close evolutionary relationships among these sequences, many of which are identical, it is possible that there was direct viral transmission among some of the travelers.

### DENV-3

In the case of DENV-3 only three sequences were obtained from travelers entering Western Australia, all of which fell into genotype I that has been described in a variety of locations across South-East Asia (Fig. 4). However, all these viruses were singletons representing phylogenetically distinct lineages within the genetic diversity of DENV-3, such that they represent three independent introductions in 2010. In all cases, the travelers in question were entering from Bali, and the closest sequences matches were viruses from Indonesia, again suggesting that the travelers have been infected by locally circulating lineages.

### DENV-4

Our sample of DENV-4 comprised nine sequences from travelers entering Western Australia, and which provided evidence for five phylogenetically distinct lineages, and hence introductions (Fig. 5). All these DENV-4 sequences fell as relatively closely related lineages within genotype III that is commonly sampled within South-East Asia. In addition, all the sequences from travelers to Western Australia were most closely related to background viruses sampled from Bali and other locations within Indonesia, indicating that these are local variants that likely evolved *in situ*. Also of note were the observations that lineage 2, which comprised four sequences, was sampled between May 2010-September 2011 such that it likely persisted within Bali for over a year, and that lineage 3 (sampled between October-November 2010) spread to both Bali and Java.

## Discussion

Dengue is endemic throughout Southeast Asia, a region where the incidence of both dengue fever and severe dengue has increased dramatically since the 1950s when the hemorrhagic form of the disease was first recognized. We analyzed DENV derived from travelers entering Western Australia after visiting seven countries throughout Asia between 2010–2012 and identified a diverse range of DENV genotypes and lineages within all four serotypes. Importantly, one of these lineages (DENV-2, Cosmopolitan genotype, lineage 4) appears to have emerged during a major outbreak in Bali in 2011–2012, and hence should be closely monitored.

Most of the travelers studied here had entered from Bali, Indonesia, a popular holiday destination for residents of Western Australia. In recent years new budget airlines began offering affordable Bali package holidays and travel increased 300-fold between 2006 and 2010, coinciding with a sharp increase in dengue cases notified to the Communicable Diseases Division of the WA Department of Heath [20]. Epidemic dengue hemorrhagic fever was first described in Indonesia (Java) in 1968 [21], and transmission of all four DENV serotypes was described during an epidemic of severe dengue focused in Jakarta (Java) in 2004, with DENV-3 the predominant serotype [22]. Genome sequence analysis of a subset of isolates showed that the DENV circulating in Indonesia were local strains with strong epidemic potential which had circulated in the region for decades [23]. In our analysis, many of the DENV1–4 sampled from Indonesia during 2010–2012 also appeared to be local regional variants, although with clear lineage importation including the presence of two DENV-1 Genotype II viruses sampled from travelers entering WA from Bali but which were most closely related to viruses circulating in China.

Bali is a major travel destination and attracts visitors from many countries, predominantly from the Asia-Pacific and ASEAN regions where dengue burden is the highest globally. The continuing annual growth increase in visitor arrivals into Bali indicates there is a high likelihood that novel DENV lineages will continue to be introduced into Bali. Official surveillance data for Bali are not immediately available. However, local newspapers reported that the Bali Health Agency recorded almost 6,000 cases in 2013 (a prevalence of 145 per 100,000 population) with a mortality rate of 1%, for a population of 4.1 million, and that there was a large number of dengue cases in 2010 with a prevalence of 365 persons per 100,000 population [24]. Our analysis indicates that there was hyperendemic transmission of all four serotypes in Bali during 2010, and circulating DENV included dominant local strains which had circulated for several years, as well as strains more recently introduced into Bali from other countries in the region. Hence, these data suggest that Bali is a melting pot of substantial DENV diversity, and therefore may in turn serve as a hub for dengue transmission and mixing. Indeed DENV traffic into other countries via travelers returning from Bali has been described [25,26]

Systematic surveillance in acutely ill travelers entering Australia from endemic Asia-Pacific countries, which are popular travel destinations, provides valuable and timely information on DENV lineages circulating in the region. We identified an emerging lineage of DENV-2 that appeared to be associated with a major outbreak in Bali in 2012, and which was also identified in travelers entering from Timor-Leste and Thailand. It is unclear how long this lineage has been present in the region as there is a dearth of publicly available contemporaneous DENV sequence data. Additional DENV-2 isolates from 2010–2014 are currently being sequenced to determine if this virus was present in other countries prior to 2012, and to confirm continued transmission after this time.

More generally, this study demonstrates that surveillance of returned travelers may provide important information on DENV genotypes and lineages circulating in dengue endemic countries where locally-generated detailed genetic data may not be available.
